# *Anopheles stephensi*: a new and important menace to South America?

**DOI:** 10.1590/0037-8682-0307-2025

**Published:** 2026-02-09

**Authors:** Raquel Miranda Gleiser, Carlos Brisola Marcondes, Maria de Fátima Freire de Melo Ximenes

**Affiliations:** 1 Universidad Nacional de Córdoba (UNC). Faculdad de Ciencias Exactas, Físicas y Naturales, Instituto Multidisciplinario de Biología Vegetal (IMBIV), Centro de Relevamiento y Evaluatión de Recursos Agrícolas y Naturales (CREAN), Córdoba, Argentina.; 2 Universidade Federal de Santa Catarina, Florianópolis, SC, Brasil.; 3 Universidade Federal do Rio Grande do Norte, Natal, RN, Brasil.

**Dear Editor**

***Anopheles stephensi*: a new and important menace to South America?**

Over the past two decades, significant progress has been made in reducing the burden of malaria in South America. Most cases are currently concentrated in the Bolivarian Republic of Venezuela, Colombia, and Brazil, while Paraguay, Argentina, El Salvador, and Suriname have been certified as malaria-free for autochthonous transmission since 2018, 2019, 2021, and 2023, respectively^1^ ^,^ ^2^. Nevertheless, sustained efforts are required to address the underlying factors that hinder disease elimination and prevent the reintroduction of malaria into areas where it has been successfully controlled. 

Although the most dangerous species, *Plasmodium falciparum*, has recently become very rare in South America (72% of cases were due to *Plasmodium vivax* in 2023), it remains more prevalent in countries such as Colombia^1^. The primary vectors, including *Anopheles darlingi Anopheles aquasalis, Anopheles deaneorum, Anopheles cruzii,* and members of the *albitarsis* complex, are mostly associated with rural and sylvatic environments, and are rarely established in urban settings.

A historical precedent of invasive malaria vectors in Brazil was the introduction of *Anopheles gambiae* s.l. (in reality *An. arabiensis*) in the northeast of the country, which silently spread in the 1930s, causing severe epidemics of malaria^3^. Its eradication in the 1940s offered valuable lessons for current vector control strategies. The key elements of this effort included early detection, swift government action, and coordinated international support. These experiences highlight the importance of proactive and sustained responses to prevent the establishment of invasive malaria vectors. More recently, *Anopheles stephensi*, originally native to South Asia and the Arabian Peninsula, invaded Africa and was first detected in Djibuti. It now occurs in multiple countries across the continent, mostly in the northeastern and eastern regions, but also in Nigeria and Niger^4^. Genetic analysis supports the idea that populations in the Horn of Africa were introduced directly from South Asia, rather than via the Arabian Peninsula^5^. 

The recent establishment of faster and more direct maritime routes connecting Brazilian ports with South Asia (e.g., Portal Portuario 2025^6^) increases the risk of accidental introduction of mosquito vectors to the American continent. Moreover, there is evidence that *An. stephensi* eggs may survive without water under optimal conditions, potentially aiding their spread through trade and transport networks^7^. To mitigate this risk, targeted entomological surveillance and vector control protocols such as inspection of water-holding containers, larval sampling, and larvicide application in high-risk ports and airport areas should be implemented. Training port personnel to identify mosquito eggs and larvae along with rapid response mechanisms can further strengthen containment efforts. Risk modeling studies have identified potential areas that can be invaded, including the Northeast Brazilian region. Over the coming decades, much of Africa and large areas in South and Central America could also become suitable habitats for this anopheline^8^.

The intense scientific focus on *An. stephensi,* as evidenced by nearly 20,000 scientific papers published over the last 15 years, with 686 appearing in 2025 alone in Google Scholar (2025), reflects its significance in global vector-borne diseases. This species comprises three forms (type, *misorensis*, and an intermediate form), distinguished by the morphology of the ridges on egg floats and molecular markers. The type form usually develops in artificial containers and has the highest vectorial capacity^9^. 

Although malaria has traditionally been a rural disease in Africa, the introduction of *An. stephensi* has boosted urban transmission^8^, particularly in sub-Saharan cities, where approximately 40% of the population lives in precarious conditions. Its overlap with *Aedes aegypti*, a species that has been impossible to eradicate, raises additional public health concerns owing to the increased risk of multiple disease outbreaks. In addition, this overlap may intensify the transmission of arboviruses, such as dengue, Zika, and chikungunya, especially in densely populated areas with inadequate sanitation. Shared larval habitats and potential insecticide resistance complicate control efforts, underscoring the need for integrated vector management strategies that simultaneously address both species. One mitigating factor is that during the dry season, larval populations tend to concentrate in a few highly productive habitats, which presents a strategic opportunity for targeted larval source management^10^. Targeting these “super-productive” habitats with larvicides or source reduction interventions may significantly reduce local populations and interrupt transmission cycles.

However, the forms in Africa are yet to be fully characterized. Notably, *An. stephens*i is more tolerant to extreme temperature conditions and does not share habitats with native vector species, such as *An. gambiae s.s.* and *Anopheles funestus* ^11^. Given its ecological traits and invasive history, its arrival on the American continent is a matter of time. To counter this emerging threat, proactive surveillance, capacity building, and effective international cooperation are urgently required. Such cooperation should include harmonized monitoring protocols, cross-border data sharing, and joint training initiatives. In Brazil, health authorities must remain alert to the possibility of urban malaria cases that are currently restricted to rural areas in the Amazonian region. Vigilance in ports, airports, and urban centers, including during the dry season when desiccation-resistant eggs may persist, is essential for detecting and containing potential introductions. 
